# K-XMSS and K-SPHINCS^+^: Enhancing Security in Next-Generation Mobile Communication and Internet Systems with Hash Based Signatures Using Korean Cryptography Algorithms

**DOI:** 10.3390/s23177558

**Published:** 2023-08-31

**Authors:** Minjoo Sim, Siwoo Eum, Gyeongju Song, Yujin Yang, Wonwoong Kim, Hwajeong Seo

**Affiliations:** 1 Department of Information Computer Engineering, Hansung University, Seoul 02876, Republic of Korea; alswntla@hansung.ac.kr (M.S.); smile267@hansung.ac.kr (S.E.); thdrudwn98@hansung.ac.kr (G.S.); 2 Department of Convergence Security, Hansung University, Seoul 02876, Republic of Korea; yangyu7@hansung.ac.kr (Y.Y.); dnjsdndeee@hansung.ac.kr (W.K.)

**Keywords:** XMSS, SPHINCS^+^, Korean cryptography algorithms, hash based signatures, software implementations

## Abstract

As Mobile Communication and Internet Systems (MCIS) have rapidly developed, security issues related to MCIS have become increasingly important. Therefore, the development and research of security technologies for mobile communication and internet systems are actively being conducted. Hash-Based Signature (HBS) uses a hash function to construct a digital signature scheme, where its security is guaranteed by the collision resistance of the hash function used. To provide sufficient security in the post-quantum environment, the length of hash should be satisfied for the security requirement. Modern HBS can be classified into stateful and stateless schemes. Two representative stateful and stateless HBS are eXtended Merkle Signature Scheme(XMSS) and SPHINCS${}^{+}$, respectively. In this paper, we propose two HBS schemes: K-XMSS and K-SPHINCS${}^{+}$, which replace internal hash functions of XMSS and SPHINCS${}^{+}$ with Korean cryptography algorithms. K-XMSS is a stateful signature, while K-SPHINCS${}^{+}$ is its stateless counterpart. We showcase the reference implementation of K-XMSS and K-SPHINCS${}^{+}$ employing Lightweight Secure Hash (LSH) and two hash functions based on block ciphers (i.e., CHAM and LEA) as the internal hash function. In addition, K-XMSS and K-SPHINCS${}^{+}$ using Advanced Vector Extensions 2 (AVX2) have been provided, demonstrating that they can be optimized for better performance using advanced implementation techniques than previous approaches.

## 1. Introduction

Recently, Internet technologies and mobile communication have made rapid progress [1]. However, this technological revolution has created various security threats to ensure the reliability and safety of MCIS [2,3,4,5,6]. In order to prevent these security threats, research is being actively conducted to propose a digital signature protocol that has been proven to be safe and efficient using hash functions that have been implemented in mobile devices [7]. Additionally, emerging technologies such as Quantum Computing, and Generative Artificial Intelligence(AI) are creating new complex security challenges. As a result, research on MCIS security, including new technologies, is actively being conducted.

HBS [8] schemes guarantee the security with collision resistance of the hash function used. HBS schemes are signature schemes that rely solely on the existence of a secure one-way function (i.e., hash function). HBS schemes were developed in the 1970s by Lamport [9] and extended by Merkle [10]. As the threat to quantum computers increases, interest in the field is also on the rise. The hash function can respond to the threat of quantum computers by increasing the output length [11]. Shor algorithm [12] is an algorithm capable of efficient prime factorization, and Grover algorithm [13] has strong search capabilities. For this reason, HBS schemes have attracted attention, and XMSS has proven the feasibility of HBS. Recently, stateless SPHINCS [14], a variant of XMSS that does not need to maintain state, has been proposed. For the the American National Institute of Standards and Technology (NIST) Post-Quantum Cryptography(PQC) standardization project [15], SPHINCS${}^{+}$, an improved version of SPHINCS, has been proposed. SHA2, SHAKE, and HARAKA hash functions were used in the implementation of XMSS and SPHINCS${}^{+}$. In this paper, we show variants of XMSS and SPHINCS${}^{+}$ (i.e., K-XMSS and K-SPHINCS${}^{+}$) by replacing the current hash function setting with Korean cryptography algorithms (i.e., LSH hash function and hash function based on Korean block ciphers). Korean cryptography algorithm means to a cryptography algorithm developed in Korea and designated as a Korean standard.

The remainder of this paper is structured as follows. Section 2 describes XMSS, SPHINCS${}^{+}$, and hash function based on block cipher. Section 3 describes the proposed implementation method. Section 4 shows the performance comparison of XMSS and K-XMSS, and the performance comparison of SPHINCS${}^{+}$ and K-SPHINCS${}^{+}$. Finally, Section 5 describes the conclusion of this work and future plan.

### 1.1. Contributions

#### 1.1.1. First Implementation Korean Version of XMSS and SPHINCS${}^{+}$

To the best of our knowledge, this is the first trial to implement Korean version of hash-based cryptography schemes with Korean hash functions. The original XMSS produces HBS through the use of SHA2 and SHAKE hash functions. The original SPHINCS${}^{+}$ produces HBS using the SHA2, SHAKE, and HARAKA hash functions. In response, we proposed to generate HBS using Korean hash functions (i.e., CHAM, LEA and LSH). As the result of evaluation performance, LSH showed the best performance among Korean hash functions in K-XMSS and K-SPHINCS${}^{+}$. In addition, K-XMSS and K-SPHINCS${}^{+}$ using AVX2 have been provided, demonstrating that they can be optimized for better performance using advanced implementation techniques such as NEON. Our Implementation is available in the public domain at https://github.com/minjoo97/K-XMSS-K-SPHINCS-project (accessed on 21 August 2023).

#### 1.1.2. Hash Function Based on Korean Block Cipher

We implemented a hash function using Korean block ciphers. Tandem DM scheme was applied to use the Korean block cipher as a hash function. Tandem DM can generate a hash value having a length of 2m-bit by applying a block cipher algorithm using an *m*-bit block length and a 2m-bit key length. In this approach, we implemented hash functions using Korean block ciphers by applying CHAM and LEA Korean block ciphers.

### 1.2. Extended Version of MobiSec’22

The work presented in Mobisec’22 is revisited in this paper. K-XMSS and K-SPHINCS${}^{+}$ utilizing reference c was presented in https://www.mobisec2022 [16] (accessed on 21 August 2023). In this paper, K-XMSS and K-SPHINCS${}^{+}$ showed that AVX2 can utilize advanced implementation techniques to optimize for better performance using advanced implementation techniques such as NEON.

## 2. Related Works

### 2.1. eXtended Merkle Signature Scheme (XMSS)

**Figure 1:**
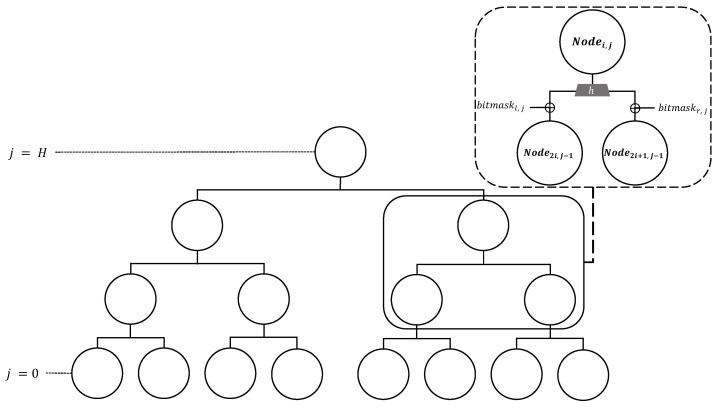
Tree structure of XMSS; *H* is height of the tree; Bitmask is chosen uniformly at random from $\left(b_{l,j}\|b_{r,j}\in {\left\{0,1\right\}}^{2n}\right)$.

XMSS [17] is a stateful HBS scheme based on the Merkle Signature Scheme (MSS) [18], and uses WOTS${}^{+}$ [19] as the main building block. XMSS uses one key pair (i.e., private key and public key), and the tree height is *H*. XMSS can generate up-to $2^{H}$ signatures, which is illustrated on Figure 1. To ensure the security of XMSS, the used key pair (i.e., WOTS${}^{+}$ key) should not be used again.

Definitions of parameters used in XMSS are given in Table 1.

#### 2.1.1. Winternitz One Time Signature (WOTS)

WOTS scheme [18,20] efficiently signs the message digest. The private key is used for signing, where the key should not be used again. In other words, it is infeasible to sign more than one message using a single private key. The signature size of WOTS is smaller than Lamport OTS [9], because the message digest is signed at the same time. WOTS is based on security against collision resistance of one-way hash function. In the signature of WOTS, *w* representing the number of bits to be signed, is used as a number of 2 or more. The signature key consists of a randomly selected *l*-bit string of length *n*, where *l* is computed as Equation (1). Equation (1) is calculated based on the selected *w*.

(1) $$l=l_{1}+l_{2},\;l_{1}=\left\lceil \frac{m}{\log_{2}\left(w\right)}\right\rceil ,\;l_{2}=\left\lfloor \frac{\log_{2}\left(l_{1}\left(w-1\right)\right)}{\log_{2}\left(w\right)}\right\rfloor +1$$

The *m*-bit message *M* is based on *w*, and the checksum *C* for the message is calculated as Equation (2).

(2) $$C=\sum_{i=1}^{l_{1}}\left(w-1-M_{i}\right)\;$$

The hash function chain of WOTS is given in Equation (3). Using the signature key *l* as an input to the function chain, we get the public key of WOTS as a result of Equation (3).

(3) $$c^{i}\left(x\right)=h_{k}\left(c^{i-1}\left(x\right)\right)=h_{k}\circ h_{k}\circ \cdots \circ h_{k}\circ h_{k},\;x\in {\left\{0,1\right\}}^{n},\;c^{0}\left(x\right)=x$$

#### 2.1.2. Winternitz One Time Signature Plus (WOTS${}^{+}$)

WOTS${}^{+}$ [19] is a descendent of WOTS scheme. WOTS${}^{+}$ increases the security by adding a random value, *r*, as shown in the process of applying the one-way function *h* in the Winternitz one-time signature technique. The function chain of WOTS${}^{+}$ is expressed by the following Equation (4). Unlike WOTS, WOTS${}^{+}$ is based on the security against secondary pre-image resistance.

(4) $$c^{i}\left(x\right)=h_{k}\left(c^{i-1}\left(x\right)⊕r_{i}\right)=\left(h_{k}⊕r_{i}\right)\circ \left(h_{k}⊕r_{1}\right)\circ \cdots \circ \left(h_{k}⊕r_{i-2}\right)\circ \left(h_{k}⊕r_{i-1}\right)$$

**Figure 2:**
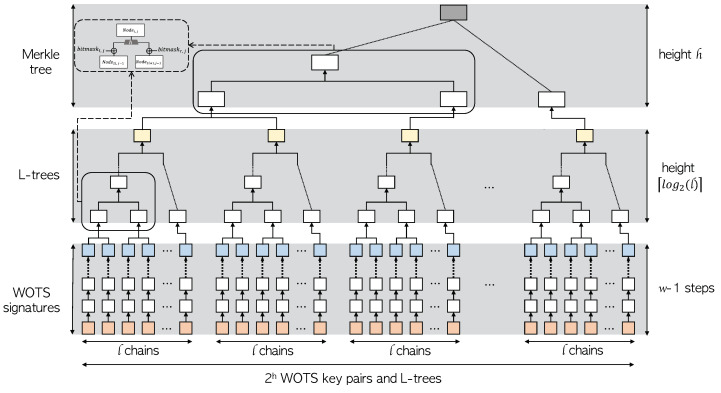
Structure of XMSS; Orange block indicates WOTS${}^{+}$ signature key, blue block indicates WOTS${}^{+}$ public key, yellow block indicates L-tree root, and gray block indicates XMSS public key.

XMSS uses a Merkle hash tree of height *h* and a binary L-tree of height  $\left\lceil {log}_{2}l\right\rceil$  to reduce the size of the public key. Two trees are used to reduce $2^{H}$ WOTS${}^{+}$ verification keys to one XMSS public key. The overall structure can be found in Figure 2. The public key of WOTS${}^{+}$ obtained using WOTS${}^{+}$ as described above constructs the leaf of the L-tree, which is an unbalanced binary tree. If there is no power of 2 leaves, a node without a right sibling is moved up until it becomes a right sibling of another node. In the case of the L-tree, the same structure as in Figure 1 is used. However, a bitmask different from that of the Merkle tree is used. The upper leaf node of the L-tree created in this way becomes the lower leaf node of the Merkle hash tree. As a result, the root node of the Merkle tree becomes the final XMSS public key. The bit length of this XMSS public key is $2(H+\left\lceil {log}_{2}l\right\rceil +1)n$, the signature length of XMSS is (l+H)n, and the private key of XMSS is less than 2n.

### 2.2. SPHINCS${}^{+}$

SPHINCS${}^{+}$ [21] is a stateless hash-based signature framework that improves the speed and signature size of SPHINCS [14]. The main contribution of SPHINCS${}^{+}$ is the introduction of FORS (i.e., few-time signature scheme). The second contribution is the method of selecting leaf nodes. SPHINCS${}^{+}$ uses functions with cryptographic properties and each parameter is defined as follows:

- *h*, *d*: parameters of Hyper-Tree
- *b*, *k*: parameters of FORS
- *w*: parameter of Winternitz

**Figure 3:**
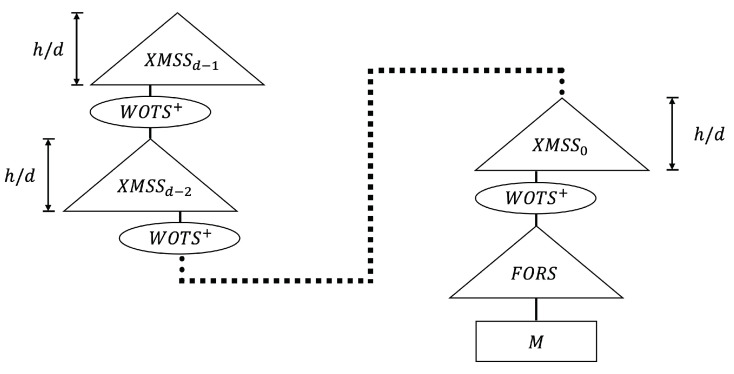
Overview of SPHINCS${}^{+}$ structure.

SPHINCS${}^{+}$ with specific parameters (*n* = 192, *h* = 51, *d* = 17, *b* = 7, *k* = 45, and *w* = 16) showed 25% shorter signatures and 1.7× faster signature routines than those of SPHINCS${}^{+}$. The structure of SHPINCS${}^{+}$ is shown in Figure 3. SPHINCS${}^{+}$ is a hyper-tree of height *h* and consists of *d* tree. The height of each tree is h/d, where *d* is involved in the signature time and the signature size. In a hyper-tree, layer(d−1) has a single tree and layer(d−2) has $2^{h/d}$ trees. The root of the layer(d−2) tree is signed using the WOTS${}^{+}$ key pair in the layer(d−1) tree. Key pairs of Layer 0 WOTS${}^{+}$ are used to sign the FORS public key. Internal values are determined through seed and bitmask, and the entire structure is not computed. For this reason, it is referred to as a “virtual structure”. More information on SPHINCS${}^{+}$ can be found in [21].

#### 2.2.1. FORS: Forest of Random Subsets

**Figure 4:**
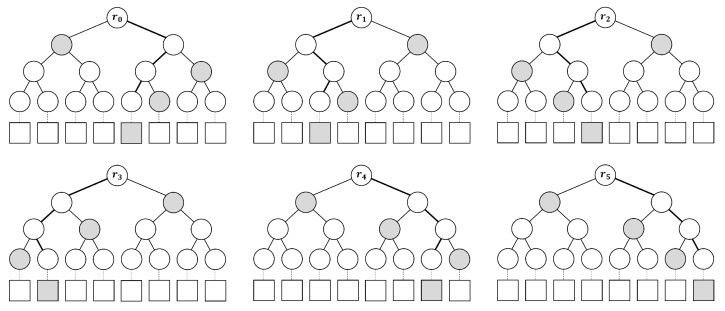
Hash tree of FORS with k=6 and a=3 for message {100 010 011 001 110 111}.

SPHINCS${}^{+}$ defines and uses FORS, a few-time signature improved from HORST [21]. FORS is defined in terms of integer *k*, t=2a and is used to sign ka-bit string. The private key of FORS consists of kt random bit values and is divided by *k* set of *t* values. Overall, it is deterministically derived from SK.seed using the pseudo-random function(PRF) and the key address of the hypertree. To obtain the FORS public key, *k* binary hash trees are constructed on the set of private key elements. Each *t* value is used as a leaf node and *k* binary hash trees with height *a* are created. Figure 4 shows the hash tree of FORS with k=6 and a=3 for message (100 010 011 001 110 111). FORS uses *H*, which is addressed using the location of the FORS key pair and the location of the function call within the tree. With WOTS${}^{+}$, the root node compresses using the Tweakable hash function ($Th_{k}$). Th is an efficient function that maps α-bit message *M* to λ-bit hash value MD using a function key of public parameter *P* and tweak *T*, and is expressed as Equation (5). The FORS public key is an *n*-bit value. The signing process of FORS is as follows. Given a message of ka bits, the *k* string of *a* bits is extracted. This bit string *k* has the index of each single leaf node of FORS. The signature consists of these nodes (indexes) and authentication paths (See Figure 4).

(5) $$\begin{array}{c} Th:P\times T\times {\left\{0,1\right\}}^{\alpha }\to {\left\{0,1\right\}}^{\lambda },\\ Th(P,T,M)=H(P||T||M) \end{array}$$

The verifier validates the public key by reconstructing the root using the certification path. Since the public key is used as a message, it is implicitly verified with the WOTS${}^{+}$ signature.

#### 2.2.2. XMSS${}^{MT}$

XMSS${}^{MT}$ [22] is an extension of XMSS. The original XMSS scheme has a disadvantage in key generation. When the height (*H*) of the tree exceeds 20, the execution time could be slow. To accelerate the performance, XMSS${}^{MT}$ uses a multi-layered XMSS tree called a hyper-tree. A hyper-tree consists of 2 or more XMSS trees, and all XMSS trees have the same height. The tree of the lowest layer of the hyper-tree is used to sign the actual message. The rest of the tree is used to sign the root node of the XMSS tree in each layer.

### 2.3. Hash Function

The hash function is used by XMSS and SPHINCS${}^{+}$ to construct the signature schemes. In this subsection, we briefly describe the LSH hash function and another hash function based on Korean block cipher [23].

#### 2.3.1. LSH Hash Function

**Figure 5:**
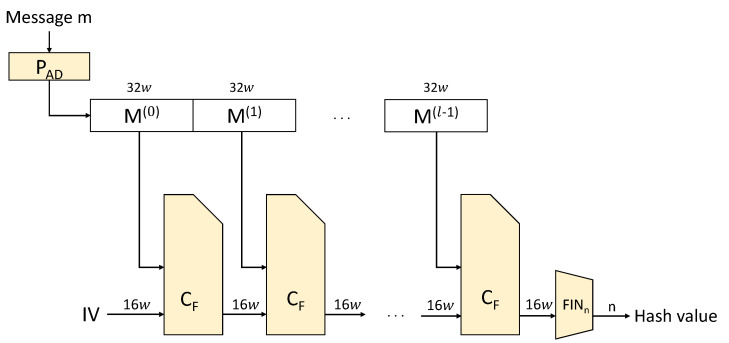
Structure of LSH hash function.

LSH is a high-speed hash function developed in Korea that generates a hash value through initialization, compression, and completion processes. The initialization process padding the message and separating the message by the size of the block. The compression process digests the message through message expansion, addition, mixing, and word-by-word circulation functions. The completion process outputs the result of the compression process as a hash value of a specific length. Figure 5 shows the operating structure of LSH.

#### 2.3.2. Hash Function Based on Block Cipher

An iterated hash function is determined by an easily computable function *h*(·,·). The function *h* is called hash round function. The input message is divided into block sizes, and the hash round function calculates the next hash value using the divided message block and the previous hash value. The number of iterations of the hash round function is repeated by the number of message blocks to obtain the final hash value. Equation (6) is a modification of the iterative hash function.

(6) $$H_{i}=h\left(H_{i-1},\;M_{i}\right)\;\;i=1,2,..n.$$

**Figure 6:**
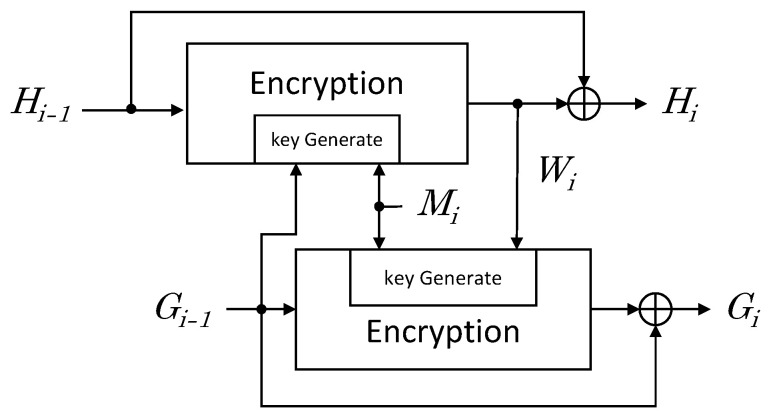
2m-bit hash round function based on *m*-bit block cipher with 2m-bit key.

Hash function based on block cipher uses a block cipher algorithm instead of a hash round function [24]. Several structures have been proposed to output the desired length of hash. We utilized the *Tandem DM* structure to implement hash functions using block ciphers. *Tandem DM* structure applies a block cipher algorithm using a key length of 2m-bit when the block length is *m*-bit, and the output hash length is 2m-bit. Figure 6 shows the *Tandem DM* Scheme.

In each iteration, two *m*-bit values (G${}_{i}$ and H${}_{i}$) are computed from the previous values H${}_{i-1}$ and G${}_{i-1}$ and from an *m*-bit message block M${}_{i}$ as follows:

(7) $$W_{i}=E_{G_{i-1},M_{i}}\left(H_{i-1}\right)$$

(8) $$H_{i}=W_{i}⊕H_{i-1}$$

(9) $$G_{i}=G_{i-1}⊕E_{M_{i},W_{i}}\left(G_{i-1}\right)$$

In this paper, *LEA* and *CHAM* block cipher algorithms were used for the hash round function.

- LEA Block Cipher LEA is a lightweight block cipher developed in Korea in 2013 to provide confidentiality not only in high-speed environments (e.g., big data and cloud), but also in lightweight environments, (e.g., IoT devices and mobile devices) [25]. The algorithm structure of LEA uses the ARX structure, and encryption proceeds by dividing the input block into four 32-bit. The ARX structure uses Addition, Rotation, and XOR operations.
- CHAM Block Cipher CHAM is a lightweight block cipher announced in ICISC’17 [26]. Subsequently, the revised version of the CHAM Block cipher was announced in ICISC’19 [27]. The revised CHAM differs from the original CHAM only in the number of rounds, and the other specifications are identical. The CHAM has different operations of odd rounds and even rounds. The CHAM of the generalized 4-branch Feistel structure is based on ARX operations.

## 3. Proposed Method

### 3.1. Hash Function Based on Block Cipher

In this paper, we construct hash function based on the Tandem DM scheme and utilize LEA and CHAM as the underlying block ciphers. Tandem DM scheme and block ciphers are described in Section 2.3. Algorithm 1 is a description for Figure 6.

The process of Algorithm 1 is as follows. The message received as input is divided into block size to proceed by the number of iterations. The iteration is repeated by the message length divided by the block size. In this paper, the message length is assumed to be a multiple of the block size. Lines 4 and 7 perform key initialization. In line 4, $G_{i}$ for the upper bit and M[i] for the lower bit are used as a key. In line 7, M[i] for the upper bit and *W* for the lower bit are used as a key. The initialized key generates a roundkey to be used for encryption through the Roundkeygenerate function. Then, $H_{i}$ and $G_{i}$ generate an encrypted value through an Encryption function, and finally XOR with *W* and $G_{i}$. If the CHAM and LEA algorithms are applied to the Roundkeygenerate function and Encryption function, hash values can be obtained through LEA and CHAM block ciphers.
**Algorithm 1** Tandem DM scheme of hash function based on block cipher**Input:** *M* (Message), ML (Message Length) **Output:** Hash value
 1:n = Block size 2:**for** i=0 to ML/n **do** 3:   M[i]: Size of Block size 4:   $Key\leftarrow G_{i},M\left[i\right]$ (if $G_{0}$, use a initialization Vector) 5:   RK←RoundKeyGenerate(Key) 6:   $W\leftarrow Encrytion(H_{i},RK)$ (if $H_{0}$, use a initialization Vector)   7:   Key←M[i],W 8:   RK←RoundKeyGenerate(Key) 9:   $TEMP\leftarrow Encrytion(G_{i},RK)$ (if $G_{0}$, use a initialization Vector)  10:   $H_{i+1}\leftarrow H_{i}⊕W_{i}$11:   $G_{i+1}\leftarrow G_{i}⊕TEMP$12:**end for** 13:**return** Hashvalue←H,G


### 3.2. K-XMSS

In this paper, we replaced the hash functions used in the original XMSS to Korean cryptography algorithms. We developed the code based on the basic C reference provided by [28].

Since XMSS has a tree height of *h* (10, 16, and 20), which determines the number of signatures with one key pair, K-XMSS adopted same parameters and structures utilized in XMSS. LSH provides the value of *n* only for 256 and 512. The hash function based on CHAM and LEA provides the value of *n* only for 256. In other words, Korean hash functions do not support the *n* value of 192, in K-XMSS. K-XMSS is performed on security parameters *n* of 256 and 512. Since the Winternitz parameter *w* of the original XMSS is fixed to 16, the value of *w* is also fixed to 16.

Functions used in K-XMSS are organized as follows:

- **F**: Key encryption hash function; **F** accepts and returns byte strings of length *n* using keys of length *n*.
- **H**: Encryption hash function; **H** accepts *n*-byte keys and byte strings with a length of 2n and returns an *n*-byte string.
- **H**${}_{msg}$: Encryption hash function; **H**${}_{msg}$ accepts 3n-byte keys and byte strings of arbitrary length and returns *n*-byte strings.
- **PRF**: Pseudo-random function; **PRF** has an *n*-byte key and a 32-byte index as input and generates pseudo-random value (length *n*).
- **toByte(x, n)**: *n*-byte string contains a binary representation of *x* (in the order of big-endian bytes);

Parameters used in K-XMSS are organized as follows:

- **KEY**: Keys with length in bytes.
- **M**: Strings with length in bytes.

For the n=32 setting, K-XMSS uses Equations (10)–(12) for LSH-256, CHAM, and LEA, respectively. For the n=64 setting, K-XMSS use Equation (13) for LSH-512.

#### 3.2.1. K-XMSS_LSH256

Following equation describes LSH256 function for K-XMSS.

(10) $$\begin{array}{c} F=\mathrm{LSH}256(toByte(0,32)||KEY||M),\\ H=\mathrm{LSH}256(toByte(1,32)||KEY||M),\\ H_{msg}=\mathrm{LSH}256\left(toByte\left(2,32\right)||KEY||M\right),\\ \mathrm{PRF}=\mathrm{LSH}256(toByte(3,32)||KEY||M). \end{array}$$

#### 3.2.2. K-XMSS_CHAM

Following equation describes CHAM function for K-XMSS.

(11) $$\begin{array}{c} F=\mathrm{CHAM}(toByte(0,32)||KEY||M),\\ H=\mathrm{CHAM}(toByte(1,32)||KEY||M),\\ H_{msg}=\mathrm{CHAM}\left(toByte\left(2,32\right)||KEY||M\right),\\ \mathrm{PRF}=\mathrm{CHAM}(toByte(3,32)||KEY||M). \end{array}$$

#### 3.2.3. K-XMSS_LEA

Following equation describes LEA function for K-XMSS.

(12) $$\begin{array}{c} F=\mathrm{LEA}(toByte(0,32)||KEY||M),\\ H=\mathrm{LEA}(toByte(1,32)||KEY||M),\\ H_{msg}=\mathrm{LEA}\left(toByte\left(2,32\right)||KEY||M\right),\\ \mathrm{PRF}=\mathrm{LEA}(toByte(3,32)||KEY||M). \end{array}$$

#### 3.2.4. K-XMSS_LSH512

Following equation describes LSH512 function for K-XMSS.

(13) $$\begin{array}{c} F=\mathrm{LSH}512(toByte(0,64)||KEY||M),\\ H=\mathrm{LSH}512(toByte(1,64)||KEY||M),\\ H_{msg}=\mathrm{LSH}512\left(toByte\left(2,64\right)||KEY||M\right),\\ \mathrm{PRF}=\mathrm{LSH}512(toByte(3,64)||KEY||M). \end{array}$$

### 3.3. K-SPHINCS${}^{+}$

Similar to XMSS, we changed the hash functions used in the existing SPHINCS${}^{+}$ to Korean hash functions). Notations used in K-SPHINCS${}^{+}$ are organized as follows:

Functions used in K-SPHINCS${}^{+}$ are organized:

- **H**${}_{msg}$: Additional key hash function that can handle messages of arbitrary length.
- **PRF**: Pseudo-random function for generating pseudo-random keys.
- **PRF**${}_{msg}$: Using PRF to generate randomness for message compression.
- **F**: Second-preimage resistant, undetectable one-way function; $B^{n}\times B^{32}\times B^{n}\to B^{n}$
- **H**: Second-preimage resistant hash function; $B^{n}\times B^{32}\times B^{2n}\to B^{n}$
- **T**${}_{l}$: Weakable hash functions of the form mapping an ln-byte message M to an *n*-byte hash value md; $B^{n}\times B^{32}\times B^{ln}\to B^{n}$

Parameters used in K-SPHINCS${}^{+}$ are organized:

- **R**: Random values generated based on messages and SK.prf
- **PK.seed**: Public seed which is part of the SPHINCS${}^{+}$ public key.
- **PK.root**: Top root node which is part of the SPHINCS${}^{+}$ public key.
- **ADRS**: 32-byte value representing an address in five defined structures.
- **SK.prf**: As one of the private key elements, the value used to deterministically generate a randomized value for a randomized message hash.
- **Optrand**: Value added when making the value of *R* optionally non-deterministic.

We set the hash function parameters (i.e., *n*, *h*, *d*, *k*, *w*) used in SPHINCS${}^{+}$ to be the same in K-SPHINCS${}^{+}$. LSH is applicable to 256 and 512-bit outputs, and CHAM and LEA are applicable to 256-bit outputs. For this reason, we implement it based on hash function-256. K-SPHINCS${}^{+}$ uses Equations (14)–(16) for LSH-256, CHAM, and LEA, respectively.

#### 3.3.1. K-SPHINCS${}^{+}-$LSH256

Following equation describes LSH256 function for K-SPHINCS${}^{+}$.

(14) $$\begin{array}{c} H_{msg}\left(R,PK.seed,PK.root,M\right)=\mathrm{LSH}256\left(R||PK.seed||PK.root||M,8m\right),\\ \mathrm{PRF}(SEED,ADRS)=\mathrm{LSH}256(SEED||ADRS,8n),\\ {\mathrm{PRF}}_{msg}\left(SK.prf,Optrand,M\right)=\mathrm{LSH}256\left(SK.prf||Optrand||M,8n\right),\\ F\left(PK.seed,ADRS,M_{1}\right)=\mathrm{LSHE}256(PK.seed||ADRS||M_{1},8n),\\ H(PK.seed,ADRS,M_{1}\left|\right|M_{2})=\mathrm{LSHE}256(PK.seed||ADRS||M_{1}\left|\right|M_{2},8n),\\ T_{l}\left(PK.seed,ADRS,M\right)=\mathrm{LSHE}256\left(PK.seed||ADRS||M,8n\right). \end{array}$$

#### 3.3.2. K-SPHINCS${}^{+}-$CHAM256

Following equation describes CHAM256 function for K-SPHINCS${}^{+}$.

(15) $$\begin{array}{c} H_{msg}\left(R,PK.seed,PK.root,M\right)=\mathrm{CHAM}\left(R||PK.seed||PK.root||M,m\right),\\ \mathrm{PRF}(SEED,ADRS)=\mathrm{CHAM}(SEED||ADRS,n),\\ {\mathrm{PRF}}_{msg}\left(SK.prf,Optrand,M\right)=\mathrm{CHAM}\left(SK.prf||Optrand||M,n\right),\\ F\left(PK.seed,ADRS,M_{1}\right)=\mathrm{CHAM}(PK.seed||ADRS||M_{1},8),\\ H(PK.seed,ADRS,M_{1}\left|\right|M_{2})=\mathrm{CHAM}(PK.seed||ADRS||M_{1}\left|\right|M_{2},n),\\ T_{l}\left(PK.seed,ADRS,M\right)=\mathrm{CHAM}\left(PK.seed||ADRS||M,n\right). \end{array}$$

#### 3.3.3. K-SPHINCS${}^{+}-$LEA256

Following equation describes LEA256 function for K-SPHINCS${}^{+}$.

(16) $$\begin{array}{c} H_{msg}\left(R,PK.seed,PK.root,M\right)=\mathrm{LEA}\left(R||PK.seed||PK.root||M,m\right),\\ \mathrm{PRF}(SEED,ADRS)=\mathrm{LEA}(SEED||ADRS,n),\\ {\mathrm{PRF}}_{msg}\left(SK.prf,Optrand,M\right)=\mathrm{LEA}\left(SK.prf||Optrand||M,n\right),\\ F\left(PK.seed,ADRS,M_{1}\right)=\mathrm{LEA}(PK.seed||ADRS||M_{1},8),\\ H(PK.seed,ADRS,M_{1}\left|\right|M_{2})=\mathrm{LEA}(PK.seed||ADRS||M_{1}\left|\right|M_{2},n),\\ T_{l}\left(PK.seed,ADRS,M\right)=\mathrm{LEA}\left(PK.seed||ADRS||M,n\right). \end{array}$$

## 4. Evaluation

The implementation was evaluated on a MacBook Pro 16 with the Intel i7-9750H processor, which can be clocked up to 2.6 GHz. Implementation is carried out on the Xcode framework, and compiled using the compile option -O3 (i.e., fastest).

### 4.1. K-XMSS vs. XMSS

XMSS was evaluated using test/speed.c included in the basic C reference code provided by [28]. K-XMSS was evaluated on the same setting by changing existing hash functions to Korean hash functions. The performance evaluation of original XMSS and proposed K-XMSS can be shown in Table 2 and Table 3.

The performance evaluation of optimized K-XMSS using AVX2 can be shown in Table 4. In XMSS, the smallest of the heights is 10. Therefore, K-XMSS performed only for tree height h of 10, which determines the number of messages that can be signed with one key pair. As described in Section 3.2, only 256 and 512 are provided for the security parameter *n* of the Korean hash functions. Therefore, *n* of K-XMSS is 256 and 512, the comparison target XMSS was also measured only for *n* values of 256 and 512.

Among Korean Hash Functions, it was confirmed that LSH was significantly faster than other hash ciphers. Furthermore, it has been confirmed that LSH_10_256 is about 3 times faster than SHA2_10_256, and achieves performance similar to SHAKE_10_256. LSH_10_512 has been confirmed to be approximately three times faster than SHA2_10_512 and SHAKE_10_512. In addition, it has been demonstrated that K-XMSS can be optimized for better performance by utilizing advanced implementation techniques through AVX2.

Table 5 is a performance measurement table of Dilithium selected as the NIST PQC standard [29]. Compared to XMSS & K-XMSS, it can be seen that the performance of Dilithium is excellent.

### 4.2. K-SPHINCS${}^{+}$ vs. SPHINCS${}^{+}$

We evaluated the performance by replacing hash functions (i.e., SHAKE, SHA, and HARAKA) used in the SPHINCS${}^{+}$ with the Korean hash functions (i.e., CHAM, LSH, and LSH).

SPHINCS${}^{+}$ was evaluated based on the simple code of PQClean project (https://csrc.nist.gov/Projects/post-quantum-cryptography accessed on 21 August 2023) [15], K-SPHINCS${}^{+}$ was evaluated by changing the hash function to a Korean hash function (i.e., LSH, CHAM, and LEA) for the same code. In the case of the original SPHINCS${}^{+}$, the same comparison was made based on [Hash function]_[256]. The performance evaluation of orginal SPHINCS${}^{+}$ & K-SPHINCS${}^{+}$ can be shown in Table 6. And the performance evaluation of optimized K-SPHINCS${}^{+}$ using AVX2 can be shown in Table 7.

Among Korean hash functions, it was confirmed that LSH was significantly faster than other hash ciphers. Furthermore, it has been confirmed that LSH_256 achieves performance similar to SHA2 and is about 0.64 times faster than SHAKE and HARAKA. In addition, it has been demonstrated that K-SPHINCS${}^{+}$ can be optimized for better performance by utilizing advanced implementation techniques through AVX2.

Since K-XMSS and K-SHPINCS${}^{+}$ adopted XMSS and SHPINCS${}^{+}$ as quantum-resistant encryption standards, it is judged to provide sufficient security in the Post-Quantum environment. Compared to SPHINCS${}^{+}$ & K-SPHINCS${}^{+}$, it can be seen that the performance of Dilithium is excellent.

Table 8 is performace of XMSS, K-XMSS. SHPINCS${}^{+}$, and SHPINCS${}^{+}$.

## 5. Conclusions

We proposed K-XMSS, K-SHPINCS${}^{+}$, which changed the hash functions of XMSS and SHPINCS${}^{+}$ (i.e., SHA2, SHAKE, and HARAKA) to Korean hash functions (i.e., LSH, CHAM, and LEA). In particular, we used Korean block ciphers (i.e., CHAM and LEA) by changing them into hash functions. Finally, we evaluated the proposed K-XMSS and K-SPHINCS${}^{+}$. Internal hash functions used in K-XMSS and K-SPHINCS${}^{+}$ used reference codes from LSH. However, there was no code implemented for hash functions based on block ciphers CHAM and LEA. Therefore, in this paper, we used the CHAM and LEA hash function reference-C code we implemented. As the result of the performance evaluation, it was confirmed that among Korean hash functions, LSH was significantly faster than other hash ciphers(i.e., CHAM and LEA). In K-XMSS, it has been confirmed that LSH_10_256 is about 3 times faster than SHA2_10_256. In K-SPHINCS${}^{+}$, it has been confirmed that LSH_256 achieves performance similar to SHA2. In addition, it has been demonstrated that K-XMSS and K-SPHINCS${}^{+}$ can be optimized for better performance by utilizing advanced implementation techniques through AVX2. Therefore, K-XMSS and K-SPHINCS${}^{+}$ can be further optimized by adopting the optimal implementation code such as NEON. Since K-XMSS and K-SHPINCS${}^{+}$ adopted XMSS and SHPINCS${}^{+}$ as quantum-resistant encryption standards, it is judged to provide sufficient security in the Post-Quantum environment. Currently, since the proposed technique is based on the reference code, performance is low when encryption except for LSH is applied. Therefore, as the future work, we propose K-XMSS and K-SPHINCS${}^{+}$, which adopt the optimal implementation code using NEON.

## Figures and Tables

**Table 1:** Symbols of XMSS parameters.

Symbols	Descriptions
*w*	Winternitz parameter (*w*); Power of two; 16 in XMSS.
*l*	Length in bytes
*c*	Hash function chain
*h*	Hash function
*C*	Checksum
*m*	Length of binary message
*r*	Randomization elements; *r* = ($r_{1}$, ⋯, $r_{w-1}$)

**Table 2:** Original XMSS and K-XMSS evaluation on Intel processors. Algorithm indicates XMSS-[Hash function]_[10]_[256]. (mid: median, avg: average, cc: clock cycle).

Algorithm	Keygen		Sign		Verify	
	sec	$10^{6}$cc	$10^{6}$cc [mid]	$10^{6}$cc [avg]	$10^{6}$cc [mid]	$10^{6}$cc [avg]
LSH	1.49	3875.89	5.91	8.36	2.09	2.14
SHAKE	1.50	3893.99	5.62	8.20	2.16	2.23
SHA2	3.53	9168.19	13.54	19.13	4.62	4.63
CHAM	5.97	15,507.85	22.66	32.19	10.61	10.47
LEA	13.22	34,369.08	49.80	69.02	16.47	16.73

**Table 3:** Original XMSS and K-XMSS evaluation on Intel processors. Algorithm indicates XMSS-[Hash function]_[10]_[512]. (mid: median, avg: average, cc: clock cycle).

Algorithm	Keygen		Sign		Verify	
	sec	$10^{6}$cc	$10^{6}$cc [mid]	$10^{6}$cc [avg]	$10^{6}$cc [mid]	$10^{6}$cc [avg]
LSH	2.96	7668.84	11.57	16.21	3.83	3.95
SHAKE	6.19	16,043.76	28.40	36.00	8.07	8.15
SHA2	7.22	18,710.56	27.47	39.19	9.58	9.79

**Table 4:** Optimized K-XMSS evaluation using AVX2 on Intel processors. Algorithm indicates XMSS-[Hash function]_[10]_[n in bits]. (mid: median, avg: average, cc: clock cycle).

Algorithm	Keygen		Sign		Verify	
	sec	$10^{6}$cc	$10^{6}$cc [mid]	$10^{6}$cc [avg]	$10^{6}$cc [mid]	$10^{6}$cc [avg]
LSH_256(AVX2)	0.55	1419.14	2.14	3.01	0.90	0.95
LSH_512(AVX2)	1.36	3548.60	5.17	7.49	1.70	1.71

**Table 5:** Evaluation Crystals-Dilithium on Intel Core-i7 6600U (Skylake) [30]. (cc: clock cycle).

Algorithm		Keygen	Sign	Verify
		$10^{6}$cc	$10^{6}$cc	$10^{6}$cc
Dilithium2	Reference-C	0.30	1.36	0.33
	AVX2	0.12	0.33	0.12
Dilithium3	Reference-C	0.54	2.35	0.52
	AVX2	0.26	0.53	0.18
Dilithium5	Reference-C	0.82	2.86	0.87
	AVX2	0.30	0.64	0.28

**Table 6:** Original SPHINCS${}^{+}$ and K-SPHINCS${}^{+}$ evaluation on Intel processors. Algorithm indicates SPHINCS${}^{+}$-[Hash function]-256f-simple. (avg: average, mid: median, cc: clock cycle).

Algorithm	Keygen		Sign		Verify	
	sec [avg]	$10^{6}$cc [mid]	sec [avg]	$10^{6}$cc [mid]	sec [avg]	$10^{6}$cc [mid]
SHA2	0.007	17.62	0.156	403.56	0.005	12.34
LSH	0.007	18.45	0.174	454.15	0.004	10.48
SHAKE	0.011	27.09	0.209	520.71	0.006	14.76
HARAKA	0.010	27.09	0.251	636.09	0.007	18.01
CHAM	0.035	90.85	0.598	1560.11	0.017	43.68
LEA	0.068	171.08	1.334	3424.09	0.037	94.09

**Table 7:** Optimized K-SPHINCS${}^{+}$ evaluation using AVX2 evaluation on Intel processors. Algorithm indicates SPHINCS${}^{+}$-[Hash function]_[n in bits]. (avg: average, mid: median, cc: clock cycle).

Algorithm	Keygen		Sign		Verify	
	sec [avg]	$10^{6}$cc [mid]	sec [avg]	$10^{6}$cc [mid]	sec [avg]	$10^{6}$cc [mid]
LSH_256(AVX2)	0.002	6.06	0.051	129.54	0.001	3.49

**Table 8:** Performance public key, private key, and signature size. It can be confirmed that K-XMSS and K-SHPINCS${}^{+}$ have the same size as the original version.

Scheme	Public Key (Byte)	Private Key (Byte)	Signature Size (Byte)
XMSS_10_256	64	1373	2500
K-XMSS_10_256			
XMSS_10_512	128	2653	9092
K-XMSS_10_512			
SPINCS+_10_256	64	128	49,856
K-SPINCS+_10_256			

## Data Availability

Not applicable.
